# The HPLC–PDA Method for Simultaneous Determination of Regalosides from Bulbs of *Lilium lancifolium* Thunb. and Their Antioxidant Effects

**DOI:** 10.3390/plants13192793

**Published:** 2024-10-05

**Authors:** Chang-Seob Seo, No Soo Kim, Kwang-Hoon Song

**Affiliations:** 1 KM Science Research Division, Korea Institute of Oriental Medicine, Yuseong-daero 1672, Yuseong-gu, Daejeon 34054, Republic of Korea; csseo0914@kiom.re.kr; 2 KM Convergence Research Division, Korea Institute of Oriental Medicine, Yuseong-daero 1672, Yuseong-gu, Daejeon 34054, Republic of Korea; nosookim@kiom.re.kr

**Keywords:** HPLC–PDA, simultaneous determination, regaloside, *Lilium lancifolium*, antioxidant effect

## Abstract

*Lilium lancifolium* Thunb. is a herbal medicine that is widely used to treat inflammation and lung diseases. In this study, a simultaneous quantitative method was developed for the quality control of BLL using high-performance liquid chromatography coupled with a photodiode array detector (HPLC–PDA), and their antioxidant effects were evaluated. Eight regalosides (i.e., regaloside A, B, C, E, F, H, I, and K) were selected as marker substances and separated on a Gemini C_18_ reversed-phase analytical column by gradient elution with distilled water–acetonitrile mobile phase containing 0.1% (*v/v*) formic acid. The method was validated with respect to linearity, sensitivities (limit of detection (LOD) and limit of quantitation (LOQ)), accuracy, and precision. The antioxidant effects of the extract and each component were evaluated using the 2,2-diphenyl-1-picrylhydrazyl radical scavenging assay and 2-2′-azino-bis(3-ethylbenzothiazoline-6-sulfonic acid) assay. The coefficients of determination values used as indicators of linearity for all components were ≥0.9999. LOD and LOQ concentrations were 0.10–0.66 μg/mL and 0.29–2.01 μg/mL, respectively. The recovery was 95.39–103.925% (relative standard deviation; RSD ≤ 2.55%), and precision RSD was <2.78%. The HPLC–PDA method was applied to real samples, and all components were detected at 1.12–29.76 mg/freeze-dried g. The evaluation of antioxidant effects showed that regalosides C, E, and K exhibited significant antioxidant effects. Our knowledge will be appropriately utilized in raw material management and conducting clinical and non-clinical studies on *L. lancifolium* or herbal medicine prescriptions containing *L. lancifolium*.

## 1. Introduction

In East Asian countries such as Korea, China, and Japan, herbal medicines, referred to as traditional Korean medicine (TKM), traditional Chinese medicine (TCM), and Kampo medicine (KM), have long been used for the treatment of or protection from various diseases. These medicines can be used alone or mixed and used as a herbal preparation. A characteristic of TCM, TKM, and KM is that they can be used for various diseases because they contain numerous active agents and have few side effects [1,2,3]. However, their quality depends on environmental factors such as weather (amount of sunshine, precipitation, temperature, etc.), harvest time, and processing and manufacturing methods [4]. Therefore, the quality control of these raw materials is required to ensure efficacy and safety.

Bulbs of *Lilium lancifolium* (BLL), one of TKM, TCM, and KM, is a perennial plant belonging to the Liliaceae family [5,6]. Since ancient times, BLL has been widely used as a component of herbal medicine prescriptions such as Baihegujin and Baihewuya decoctions because it helps to relieve coughing by promoting lung energy and is effective in treating bronchitis [5,7,8,9,10,11]. Furthermore, the biological activities of BLL have been reported to include anti-inflammatory, antibacterial, antiseizure, antitumor, immunomodulatory, pro-neurogenic, and antimelanogenic effects [8,12,13,14,15,16,17,18,19,20,21]. However, research on the antioxidant effects of regalosides, the main components of BLL, is limited.

Phytochemical research on BLL has led to the isolation of a range of components such as phenylpropanoid glucosides (e.g., regaloside A, regaloside B, etc.), phenylpropanoid glycerides (e.g., 1-*O*-*p*-coumaroylglycerol, 1-*O*-feruloyl-3-*O*-*p*-coumaroylglycerol, etc.), phenolics (e.g., rutin, *p*-coumaric acid, etc.), polysaccharides (e.g., LBP-1 composed of glucose and LP2-1 composed of rhamnose, arabinose, glucose, and galactose), and steroidal saponins (e.g., lililancifoloside B, lililancifoloside C, etc.) [15,16,17,18,19,20,21].

Given that BLL has been reported to have various effects and a range of components, quality control is crucial for consistent management of the product. The quality control of TCM, TKM, and KM is currently performed using analytical equipment such as high-performance liquid chromatography (HPLC), liquid chromatography with mass spectrometry (LC–MS), gas chromatography (GC), and gas chromatography with mass spectrometry (GC–MS). Gao et al. [20] and Sun et al. [21] analyzed polysaccharide components that exhibit antioxidant, antitumor, and immunomodulatory effects using HPLC and GC. HPLC profiling analyses of phenolic and phenylpropanoid glucoside components have also been reported [12,18,19,22]. In addition, Park et al. [14] reported the presence of organic components such as glucosides, amino acids, and regaloside A, a bioactive compound unique to BLL, in *L. lancifolium* root extract using high-performance liquid chromatography–high-resolution mass spectrometry (HPLC–HRMS). However, these analytical methods have limitations in that they have only been applied for profiling analyses or quantitative analysis of BLL, and no analytical method validation has been undertaken.

In this study, a simultaneous analysis method using HPLC was developed for quality control of BLL using eight regalosides (A, B, C, E, F, H, I, and K), which are unique to BLL, and the analytical method was validated through linearity, sensitivity, accuracy, and precision. Furthermore, their antioxidant effects were evaluated using the 2,2-diphenyl-1-picrylhydrazyl (DPPH) radical scavenging assay and 2-2′-azino-bis(3-ethylbenzothiazoline-6-sulfonic acid) (ABTS) assay.

## 2. Results and Discussion

### 2.1. Optimization of HPLC Conditions for Simultaneous Determination of Regalosides

**Figure 1:**
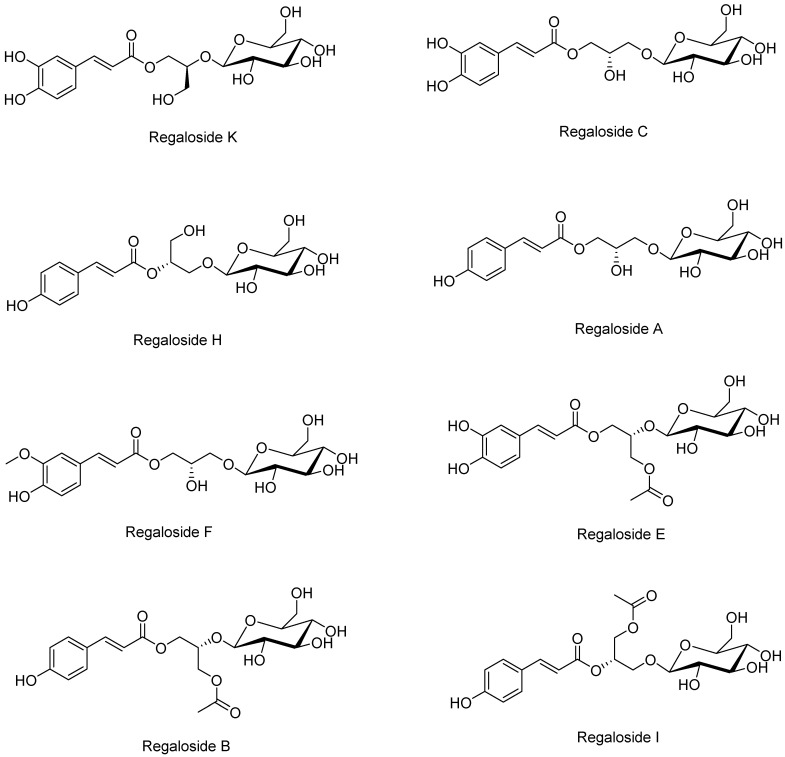
Chemical structures of the eight regalosides selected as a marker compound for quality control of BLL.

**Figure 2:**
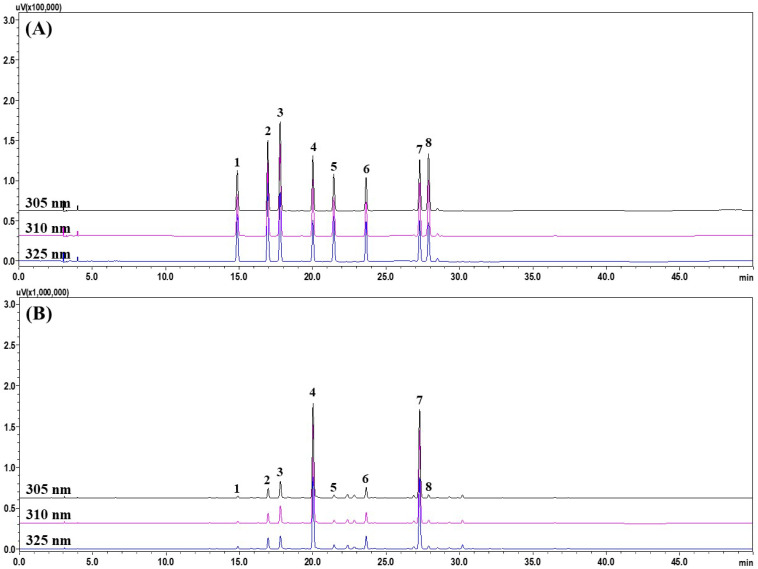
HPLC chromatograms of mixed standard compounds (**A**) and BLL extract (**B**) measured at 305, 310, and 325 nm. Regaloside K (1), regaloside C (2), regaloside H (3), regaloside A (4), regaloside F (5), regaloside E (6), regaloside B (7), and regaloside I (8).

For simultaneous analysis of the eight regalosides (Figure 1) from BLL, we tested several parameters such as type of column, addition of acid to the mobile phase, and changes in column temperature. First, we compared reversed-phased C_18_ columns (Gemini, YMC Triart, and Hypersil GOLD) from various manufacturers: Phenomenex (Torrance, CA, USA), YMC (Tokyo, Japan), and Thermo Fisher Scientific (Waltham, MA, USA). The specifications of these columns were identical: 4.6 mm inner diameter, 250 mm length, and 5 μm particle size. As shown in Supplementary Figure S1, a better resolution of regaloside B was achieved with the Gemini C_18_ column compared with the other columns. Moreover, with the YMC Triart C_18_ column, regaloside E was not completely separated from the neighboring peak (Supplementary Figure S1B). Peak resolution and patterns were then compared according to the types of acids (formic acid, trifluoroacetic acid, phosphoric acid, and acetic acid) added to the mobile phase; however, no significant difference was observed for either peak resolution or shape in the presence of the acids (Supplementary Figure S2). Finally, column temperatures (30, 40, and 50 °C) were compared, and it was found that although the resolution of peaks adjacent to regaloside A was optimal at 50 °C, the resolution of regaloside I was worse than at other temperatures (Supplementary Figure S3). To achieve the separation of both regalosides A and I, in addition to the separation of all eight regalosides, an optimal column temperature of 40 °C was established. Based on the optimization studies summarized above, the column, acid, and column temperature for simultaneous analysis of the eight regalosides were determined to be a Gemini C_18_ column, formic acid, and 40 °C, respectively. Other details of the HPLC operating conditions are presented in Supplementary Table S1. Under optimized analysis conditions, all compounds were eluted within 30 min; a representative HPLC chromatogram and UV spectra of each compound are shown in Figure 2 and Supplementary Figure S4, respectively.

### 2.2. Method Validation of the Developed HPLC Analytical Method

Method validation parameters, namely linearity (regression equation and coefficients of determination (*R*^2^)), sensitivities (limit of detection (LOD) and limit of quantitation (LOQ)), accuracy (recovery), and precision, were determined to validate the developed HPLC method. As shown in Table 1, the *r*^2^ values in the calibration curve of the eight regalosides were ≥0.9999, indicating excellent linearity. LOD and LOQ concentrations of the analytes were 0.10–0.66 μg/mL and 0.29–2.01 μg/mL, respectively (Table 1). The accuracy was 95.39–103.92% (relative standard deviation (RSD) ≤ 2.55, Table 2) as determined by recovery tests at three different concentrations. The recovery results were within the acceptable level of ±20%, confirming that the analysis method was appropriate. The precision of the developed HPLC method for analysis of the eight regalosides was tested, and the results showed that RSD values for inter- and intraday analyses and repeatability were ≤2.78, thus meeting the acceptable range of 20% (Supplementary Tables S2 and S3 and Table 3).

### 2.3. Stability of the Eight Regalosides

The stability of a sample solution of the eight regalosides was assessed at room temperature for seven days. The results showed that the concentrations of all the compounds were maintained at 94.95–103.98% (RSD ≤ 1.91) (Table 4), confirming that the eight regalosides in the sample were stable for the duration of the assessment.

### 2.4. System Suitability Test for the Developed HPLC Assay

System suitability parameters such as the retention factor (*k′*, 3.90–8.21), selectivity factor (*α*, 1.03–1.18), theoretical plate number (*N*, 266,830.64–870,983.48), resolution (*Rs*, 3.12–11.61), and symmetry factor (*S*, 1.01–1.08) (Supplementary Table S4) showed satisfactory results within the acceptable range for the developed HPLC method [23].

### 2.5. Simultaneous Determination of the Eight Regalosides in BLL

The analysis method developed for efficient quality control using major compounds (regalosides K, C, H, A, F, E, B, and I) of BLL was successfully applied to the simultaneous determination of real samples. All the components were detected at concentrations of 1.12–29.76 mg/freeze-dried g. Among them, regalosides A and B were the most abundant at 24.82–25.16 mg/g and 28.99–29.76 mg/g, respectively (Table 5).

### 2.6. Radical Scavenging Activities of BLL Extract and Regalosides

**Figure 3:**
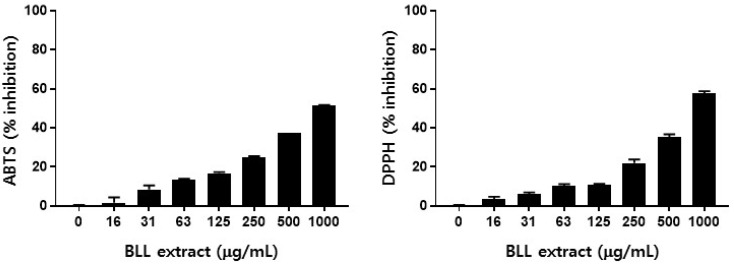
Free radical scavenging activities of BLL extract. The cationic and anionic radical scavenging activities of BLL extract were determined using ABTS and DPPH assays, respectively (*n* = 2).

**Figure 4:**
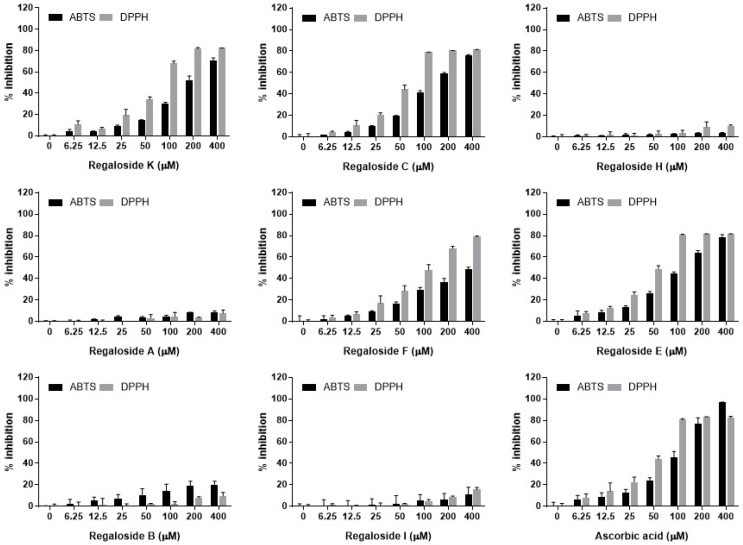
Free radical scavenging activities of the eight regalosides. The cationic and anionic radical scavenging activities of each individual chemical were determined using ABTS and DPPH assays, respectively. Ascorbic acid was used as a positive control for both assays (*n* = 2).

Oxidative stress is known to be related to various chronic disorders, including obesity, diabetes, cardiovascular diseases, degenerative brain diseases, and cancers [24,25,26,27]. Oxidative stress results from an imbalance between the generation and destruction of cellular oxidant species, and dysregulated oxidative stress leads to cellular damage through the deleterious modification of DNA, protein, and lipids [28]. Therefore, oxidative stress-related cell signaling has been suggested as a target for the treatment or prevention of oxidative stress-related disorders. In this study, we evaluated the antioxidant potentials of the BLL extract and regalosides that were identified in HPLC-assisted phytochemical analysis using ABTS and DPPH radical scavenging assays. As shown in Figure 3, the BLL extract demonstrated a dose-dependent cationic (ABTS) and anionic (DPPH) radical scavenging activity, which is consistent with previous reports [16,22,29]. The calculated IC_50_ values of the BLL extract were 942.8 μg/mL in ABTS and 813.7 μg/mL in DPPH assays (Supplementary Table S5). The radical scavenging activity assays were also applied to the eight regaloside isotypes identified as major components of the BLL extract. As shown in Figure 4, regaloside K (192. 6 μM), regaloside C (139.0 μM), and regaloside E (121.1 μM) showed remarkable ABTS radical scavenging activities. Similarly, regaloside K (66.1 μM), regaloside C (51.6 μM), regaloside F (104.5 μM), and regaloside E (46.6 μM) showed remarkable DPPH radical scavenging activities (Supplementary Table S5). Regaloside E exerted a radical scavenging activity comparable to that of ascorbic acid (108.2 μM for ABTS and 50.7 μM for DPPH), which served as a positive control in both assays. Other regalosides showed weak or no radical scavenging activities (IC_50_ > 400 μM). Along with other components of BLL, such as polysaccharides [30], these biologically active regalosides may contribute to the antioxidant activity of the BLL extract.

## 3. Materials and Methods

### 3.1. Chemicals and Reagents

Reference standards (Supplementary Figure S1) of the eight regalosides used for the quality assessment of BLL using the HPLC–PDA system were obtained from specialized suppliers of high-purity natural products: BioFron (Fullerton, CA, USA), Shanghai Sunny Biotech (Shanghai, China), Wuhan ChemFaces Biochemical (Wuhan, China), and Wuhan ChemNorm Biotech (Wuhan, China). Detailed information on these components is presented in Table S6. HPLC-grade solvents, methanol (catalog No. 9093-88, ≥99.9%), acetonitrile (catalog No. 9017-88, ≥99.9%), and water (catalog No. 4218-88) were purchased from J. T. Baker (Phillipsburg, NJ, USA). Reagents, formic acid (catalog No. 695076, 96.0%), trifluoroacetic acid (catalog No. 302031, ≥99.0%), phosphoric acid (catalog No. 49685, 85%), and acetic acid (catalog No. 1.00063, 100%) were all HPLC-grade or ACS reagent and purchased from Merck KGaA (Darmstadt, Germany). For antioxidant activity testing, ABTS (catalog No. A2166, >98.0%), DPPH (catalog No. D4313, >97.0%), potassium persulfate (catalog No. 216224, ≥99.0%), and ascorbic acid (catalog No. A4544, ≥98.0%) were obtained from Tokyo Chemical Industry (Tokyo, Japan) or Merck KGaA (Darmstadt, Germany).

### 3.2. Plant Materials and Preparation of 70% Ethanol Extract

Dried BLL was purchased from Omniherb (Uiseong, Korea) and used after morphological and sensory evaluation by Dr. Goyo Choi, a pharmacognosist (Korea Institute of Oriental Medicine, Naju, Korea). It is oblong, 2–5 cm in length, 1–2 cm in width, and 2–5 mm in thickness, and the outer surface is milky white or yellowish brown (Supplementary Figure S5). A 70% ethanol extract of BLL was prepared by KOC Biotech (Daejeon, Korea). Briefly, 70% ethanol (10 times the amount of raw material) was added, and reflux extraction was conducted at 85 °C for 3 h. After removing the organic solvent from the extract solution using a rotary evaporator (EV-1020, SciLab, Seoul, Korea), the residue was freeze-dried (LP20, IlshinBioBase, Dongduchen, Korea) and powdered. The yield was 6.33%. A voucher specimen (LBE70) of the final extract has been deposited at the Korea Institute of Oriental Medicine (Daejeon, Korea).

### 3.3. HPLC Conditions for Simultaneous Analysis of the Eight Regalosides in BLL

Simultaneous analysis of the eight regalosides in BLL was performed using a Prominence LC-20A series HPLC system (Shimadzu, Kyoto, Japan) consisting of a solvent delivery unit, online degasser, column oven, autosampler, PDA, and system controller (LabSolution, version 5.117). The eight selected regalosides were subjected to chromatographic separation on a Gemini reverse-phase column (Phenomenex, Torrance, CA, USA, 4.6 × 250 mm, 5 μm) maintained at 40 °C with gradient elution in a two-solvent system comprising water and acetonitrile (both contained 0.1% (*v/v*) formic acid). Details of the analytical conditions for simultaneous analysis of the eight regalosides in the BLL extract are provided in Supplementary Table S1.

### 3.4. Validation of the HPLC Analytical Method

The developed HPLC–PDA analytical method was validated by evaluating linearity, sensitivities (LOD and LOQ), accuracy (recovery), and precision based on the International Conference on Harmonisation guidelines, namely the methods for Q2B validation of analytical procedures [31]. Briefly, linearity was evaluated based on the *R*^2^ value of the calibration curve prepared at different concentrations of each regaloside, as shown in Table 1. LOD and LOQ values, which are measures of sensitivity validation, were calculated as follows based on the standard deviation of the response and the slope (Equations (1) and (2)):

(1) $$LOD\left(\mu g/mL\right)=3.3\times \frac{\sigma }{S}$$

(2) $$LOQ\left(\mu g/mL\right)=10\times \frac{\sigma }{S}$$

where *σ* is the standard deviation of the response, and *S* is the slope of the calibration curve.

Accuracy was assessed using a recovery test. For this, three different concentrations (low, medium, and high) of regaloside standards were added to the BLL extract, followed by ultrasonic extraction for 60 min and analysis to determine the recovery (Equation (3)).

(3) $$Recovery\left(\%\right)=\frac{found\;amount-original\;amount}{spiked\;amount}\times 100$$

For precision, intraday precision, interday precision, and repeatability were evaluated. Intra- and interday precisions were evaluated by RSD values by measuring five times each at three different concentrations (low, medium, and high) during one day and on three consecutive days. Repeatability was evaluated by RSD value by measuring six times using a standard solution mixed with the eight regalosides (Equation (4)).

(4) $$RSD\left(\%\right)=\frac{standard\;deviation}{mean}\times 100$$

### 3.5. Stability Test for the Eight Regalosides

The stability of the eight regalosides was tested at room temperature for 7 days using the sample solution. The concentration change over 7 days was compared and evaluated based on the concentration measured on the initial day (day 0) as 100%.

### 3.6. System Suitability Test

The system suitability was tested by evaluating parameters such as k′, α, N, Rs, and S using a standard solution mixed with the eight regalosides. The ranges of these parameters were set as k′ > 2, α > 1, N > 2000, Rs > 1.5, and S < 2.0, respectively.

### 3.7. ABTS and DPPH Assays

The cationic radical scavenging activities were determined using the ABTS assay. Stock solutions of ABTS (7.4 mM) and potassium persulfate (2.6 mM) were prepared in pure water. An ABTS radical solution was prepared in a dark tube by mixing equal volumes of ABTS and potassium persulfate stock solutions for 2 h. The ABTS radical solution was then further diluted with pure water to obtain an ABTS working solution with an optical density at 734 nm (OD734) of ca. 0.7. In a clear flat-bottomed 96-well assay plate (catalog No. 439454, Thermo Fisher Scientific, Rockford, IL, USA), equal volumes of serially diluted BLL extract, regalosides, or ascorbic acid were mixed with the ABTS working solution and incubated in the dark for 7 min at 22–24 °C. The decolorization by the radical scavenging activity of the test materials was optically monitored at 734 nm using the SpectraMax i3 (Molecular Devices) microplate reader (Sunnyvale, CA, USA).

The anionic radical scavenging activity was determined using the DPPH assay. A working solution of DPPH (0.2 mM) was prepared in absolute methanol in a dark tube. In a clear flat-bottomed 96-well assay plate, equal volumes of serially diluted BLL extract, regalosides, or ascorbic acid were mixed with the DPPH working solution and incubated in the dark for 30 min at 22–24 °C. The decolorization by the test materials was optically measured at 514 nm with a SpectraMax i3 microplate reader.

The radical scavenging activities of test materials in the ABTS and DPPH assays were calculated using the following formula: inhibition (%) = (1–OD of test materials/OD of control buffer) × 100. The half maximal inhibitory concentration (IC_50_) of the test material was determined with a 4-parameter logistic function using the SigmaPlot program (version. 14.5, Grafiti, Palo Alto, CA, USA). The ABTS and DPPH radical scavenging assays were performed in duplicate.

### 3.8. Statistical Analysis

Numerical data values are presented as the means ± SD of multiple experiments, and they were analyzed using GraphPad Prism v7.05 (GraphPad Software, San Diego, CA, USA). The mean differences among the experimental groups were analyzed by one-way ANOVA and post hoc Dunnett’s multiple comparison and considered statistically significant at *p* < 0.05.

## 4. Conclusions

We developed a simultaneous analysis method for evaluating the quality of BLL using HPLC coupled with a photodiode array detector (HPLC–PDA), which is simple and easy to operate and is currently the most widely used technique in the field. The developed analysis method was validated with respect to linearity, LOD, LOQ, accuracy (recovery), and precision. In a practical application of the method, it was confirmed that regalosides A and B were significantly more abundant than other components (regalosides K, C, H, F, E, and I). Furthermore, the ABTS and DPPH antioxidant activities of the BLL extract and the eight regalosides were evaluated. Regalosides K, C, and E showed higher activity compared with the other regalosides as a result of ABTS and DPPH activities. The validated method developed in this study is suitable for use in quality control protocols, efficacy studies, and clinical research and analysis.

## Figures and Tables

**Table 1:** Detection wavelength, retention times, linear ranges, regression equations, coefficients of determination (*R*^2^), limits of detection (LODs), and limits of quantitation (LOQs) for simultaneous determination of the eight regalosides.

Analyte	Detection Wavelength (nm)	Retention Time (Min)	Linear Range (μg/L)	Regression Equation (y = ax + b) ^a^	*R* ^2^	LOD (μg/L)	LOQ (μg/L)
Regaloside K	325	14.84	0.78–50.00	y = 18,555.43x + 628.08	1.0000	0.10	0.29
Regaloside C	325	16.92	1.56–100.00	y = 26,169.80x + 5579.89	1.0000	0.42	1.29
Regaloside H	310	17.76	2.34–150.00	y = 33,899.11x + 13,692.42	1.0000	0.53	1.60
Regaloside A	310	20.00	1.56–100.00	y = 33,863.89x + 8440.64	0.9999	0.20	0.60
Regaloside F	325	21.45	0.78–50.00	y = 26,477.03x + 3836.01	0.9999	0.19	0.58
Regaloside E	325	23.64	2.34–150.00	y = 23,953.18x + 8004.29	1.0000	0.66	2.01
Regaloside B	310	27.30	1.56–100.00	y = 29,530.21x + 7566.24	1.0000	0.40	1.22
Regaloside I	305	27.96	2.34–150.00	y = 11,233.13x + 4214.44	0.9999	0.57	1.72

^a^ *y*: peak area of compounds; *x*: concentration (μg/L) of compounds.

**Table 2:** Recovery test of the eight analytes with the developed analytical method.

Analyte	Original Amount (μg/L)	Spiked Amount (μg/L)	Found Amount (μg/L)	Recovery (*n* = 5)		
				Mean (%)	SD ^a^	RSD (%) ^b^
Regaloside K	11.15	2.00	13.16	100.78	1.04	1.03
		5.00	16.13	99.75	0.37	0.37
		10.00	21.33	101.85	1.08	1.06
Regaloside C	35.05	8.00	43.01	99.46	2.54	2.55
		20.00	54.96	99.53	0.47	0.47
		40.00	75.90	102.11	0.45	0.44
Regaloside H	47.78	10.00	57.60	98.16	0.96	0.98
		25.00	73.76	103.92	0.53	0.51
		50.00	98.11	100.67	0.63	0.63
Regaloside A	25.33	6.00	31.51	103.07	0.79	0.76
		15.00	39.69	95.78	0.45	0.47
		30.00	53.94	95.39	0.56	0.58
Regaloside F	14.92	3.00	17.85	97.76	1.30	1.33
		7.50	22.08	95.46	0.43	0.45
		15.00	29.41	96.57	1.42	1.47
Regaloside E	47.01	10.00	56.86	98.53	2.06	2.10
		25.00	71.74	98.96	0.69	0.70
		50.00	96.27	98.54	0.56	0.57
Regaloside B	27.93	6.00	33.74	96.89	1.08	1.11
		15.00	42.53	97.36	1.60	1.65
		30.00	58.29	101.21	0.07	0.07
Regaloside I	26.16	6.00	32.26	101.71	1.01	1.00
		15.00	40.69	96.84	0.58	0.60
		30.00	55.70	98.46	0.24	0.25

^a^ SD: standard deviation. ^b^ RSD: relative standard deviation.

**Table 3:** Precision data for the eight analytes with the developed analytical method.

Analyte	Concentration (μg/mL)	Precision (*n* = 5)					
		Intraday			Interday		
		Found Concentration (μg/mL)	Precision (RSD ^a^, %)	Accuracy (%)	Found Concentration (μg/mL)	Precision (RSD, %)	Accuracy (%)
Regaloside K	12.5	12.84	1.07	102.71	13.10	1.84	104.77
	25.0	25.55	0.91	102.19	26.04	1.85	104.18
	50.0	50.15	0.72	100.29	50.29	0.58	100.57
Regaloside C	25.0	25.55	0.71	102.21	26.03	1.77	104.13
	50.0	50.71	0.45	101.42	51.86	2.14	103.72
	100.0	99.43	0.29	99.43	100.40	0.91	100.40
Regaloside H	37.5	37.88	0.37	101.02	38.18	0.83	101.80
	75.0	74.87	0.34	99.82	75.40	0.87	100.54
	150.0	147.32	0.50	98.21	146.46	0.70	97.64
Regaloside A	25.0	25.89	1.06	103.56	26.69	2.78	106.77
	50.0	51.83	0.98	103.66	54.85	1.95	109.69
	100.0	101.57	0.43	101.57	104.20	2.25	104.20
Regaloside F	12.5	12.72	0.57	101.78	12.97	1.69	103.75
	25.0	25.26	0.41	101.06	25.69	1.66	102.76
	50.0	49.52	0.20	99.03	49.72	0.53	99.44
Regaloside E	37.5	38.22	0.62	101.92	38.84	1.54	103.57
	75.0	75.81	0.44	101.08	77.26	1.85	103.01
	150.0	148.85	0.17	99.24	149.77	0.63	99.85
Regaloside B	25.0	25.50	0.57	102.00	25.92	1.56	103.70
	50.0	50.62	0.44	101.25	51.60	1.86	103.20
	100.0	99.28	0.12	99.28	99.92	0.66	99.92
Regaloside I	37.5	38.34	0.63	102.25	38.96	1.51	103.89
	75.0	76.11	0.38	101.48	77.56	1.84	103.41
	150.0	148.76	0.13	99.17	149.73	0.67	99.82

^a^ RSD: relative standard deviation.

**Table 4:** Stability test of the eight regalosides using a sample solution at room temperature.

Analyte	Time (Day, %)						Mean	SD	RSD (%)
	0	1	2	3	4	7			
Regaloside K	100.00	96.62	95.29	95.82	94.95	97.25	96.65	1.84	1.91
Regaloside C	100.00	100.44	100.68	101.32	101.56	102.86	101.14	1.02	1.00
Regaloside H	100.00	100.01	99.44	98.88	98.68	98.40	99.24	0.68	0.69
Regaloside A	100.00	99.80	100.83	101.39	101.70	103.98	101.28	1.52	1.50
Regaloside F	100.00	98.95	98.26	98.28	99.32	101.17	99.33	1.12	1.12
Regaloside E	100.00	100.03	99.54	99.79	99.26	98.23	99.47	0.68	0.68
Regaloside B	100.00	100.04	99.31	99.31	98.43	97.33	99.07	1.03	1.04
Regaloside I	100.00	97.93	98.41	98.00	96.76	96.43	97.92	1.28	1.30

**Table 5:** Amounts of the eight regalosides measured in 70% ethanol extract of BLL with the developed analytical method (*n* = 3).

Analyte	Amount (mg/Freeze-Dried g)								
	Batch 1			Batch 2			Batch 3		
	Mean	SD ^a^ (×10^−1^)	RSD (%) ^b^	Mean	SD (×10^−1^)	RSD (%)	Mean	SD (×10^−1^)	RSD (%)
Regaloside K	1.12	0.06	0.50	1.12	0.01	0.09	1.12	0.08	0.70
Regaloside C	3.54	0.11	0.31	3.55	0.06	0.18	3.47	0.06	0.16
Regaloside H	4.67	0.05	0.12	4.66	0.09	0.18	4.68	0.11	0.23
Regaloside A	25.16	0.17	0.07	25.03	0.44	0.17	24.82	0.36	0.15
Regaloside F	1.51	0.01	0.09	1.51	0.03	0.17	1.51	0.08	0.52
Regaloside E	4.71	0.06	0.12	4.73	0.02	0.05	4.73	0.17	0.36
Regaloside B	29.19	0.41	0.14	28.99	0.12	0.04	29.76	0.11	0.04
Regaloside I	2.66	0.02	0.08	2.64	0.02	0.09	2.71	0.07	0.24

^a^ SD: standard deviation. ^b^ RSD: relative standard deviation.

## Data Availability

The data presented in this study are available in the article (tables and figures).

## Supplementary Materials

The following are available online at https://www.mdpi.com/article/10.3390/plants13192793/s1, Figure S1: Comparison of HPLC chromatograms according to column type at a column temperature of 40 °C and a mobile phase containing 0.1% (v/v) formic acid. A; Gemini C_18_ column. B; YMC-Triart C_18_ column. C; Hypersil GOLD C_18_ column. Regaloside K (1), regaloside C (2), regaloside H (3), regaloside A (4), regaloside F (5), regaloside E (6), regaloside B (7), and regaloside I (8), Figure S2: Comparison of HPLC chromatograms according to type of acid on a Gemini column maintained at 40 °C. A; 0.1% (v/v) formic acid, B; 0.1% (v/v) trifluoroacetic acid, C; 0.1% (v/v) phosphoric acid, and D; 1.0% (v/v) acetic acid. Regaloside K (1), regaloside C (2), regaloside H (3), regaloside A (4), regaloside F (5), regaloside E (6), regaloside B (7), and regaloside I (8), Figure S3: Comparison of HPLC chromatograms according to column temperature on a Gemini column and a mobile phase containing 0.1% (v/v) formic acid. A, 30 °C; B, 40 °C; and C; 50 °C. Regaloside K (1), regaloside C (2), regaloside H (3), regaloside A (4), regaloside F (5), regaloside E (6), regaloside B (7), and regaloside I (8), Figure S4: UV spectra of each regaloside, Figure S5: BLL used in the study, Table S1: Analytical conditions for simultaneous analysis of the eight regalosides in BLL sample by HPLC–PDA, Table S2: Repeatability on the retention time of each analyte (*n* = 6), Table S3: Repeatability on the peak area of each analyte (*n* = 6), Table S4: System suitability for simultaneous analysis of the eight regalosides by the HPLC method, Table S5: IC_50_ values of BLL extract and the eight regalosides in ABTS and DPPH ROS scavenging assays, Table S6: Information on the eight regalosides selected as a marker compounds for quality control of BLL.
