# Supplementary material for: The HPLC–PDA Method for Simultaneous Determination of Regalosides from Bulbs of Lilium lancifolium Thunb. and Their Antioxidant Effects
Source: Plants (Basel). 2024 Oct 5;13(19):2793. doi: 10.3390/plants13192793 (PMC11478609; doi:10.3390/plants13192793)
Supplement: Supplementary file 1 [file plants-13-02793-s001.zip › plants-3215016-Supplementary.pdf]

Table S1

Analytical conditions for simultaneous analysis of the eight regalosides in BLL sample by HPLC–PDA.

**Chromatographic parameter**			
Column	Gemini C_{18} analytical column (250 mm × 4.6 mm, 5 μm)		
Detector	PDA[a] (305, 310, and 325 nm)		
Flow rate	1.0 mL/min		
Injection volume	10.0 μL		
Column temperature	40.0 °C		
Mobile phase	Solvent A: 0.1% (v/v) formic acid in distilled water<br>Solvent B: 0.1% (v/v) formic acid in acetonitrile		
Gradient elution	Time (min)	Solvent A (%)	Solvent B (%)
	0	95	5
	30	70	30
	35	70	35
	40	95	5
	50	95	5

[a]PDA; photo-diode array detector

Table S2

Repeatability on the retention time of each analyte (n = 6).

Analyte[a]	No. (#), retention time (min)						Mean	SD	RSD (%)
	1	2	3	4	5	6			
1	14.81	14.83	14.83	14.81	14.84	14.85	14.83	0.02	0.11
2	16.89	16.91	16.91	16.90	16.92	16.93	16.91	0.01	0.09
3	17.74	17.76	17.75	17.75	17.77	17.78	17.76	0.01	0.07
4	19.98	19.99	19.99	19.99	20.01	20.01	19.99	0.01	0.06
5	21.43	21.44	21.44	21.44	21.46	21.46	21.44	0.01	0.05
6	23.62	23.63	23.63	23.64	23.65	23.65	23.64	0.01	0.06
7	27.28	27.30	27.29	27.30	27.31	27.31	27.30	0.01	0.04
8	27.94	27.96	27.95	27.96	27.97	27.97	27.96	0.01	0.04

[a]Regaloside K (1), regaloside C (2), regaloside H (3), regaloside A (4), regaloside F (5), regaloside E (6), regaloside B (7), and regaloside I (8).

Table S3

Repeatability on the peak area of each analyte (n = 6).

Analyte[a]	No. (#), peak area						Mean	SD	RSD (%)
	1	2	3	4	5	6			
1	890,623	888,842	895,268	896,057	900,328	900,752	895,311.67	4,881.77	0.55
2	1,271,855	1,269,691	1,273,595	1,277,790	1,278,832	1,283,461	1,275,870.67	5,090.36	0.40
3	1,281,676	1,277,312	1,283,622	1,285,001	1,289,437	1,286,407	1,283,909.17	4,161.26	0.32
4	1,065,420	1,063,999	1,070,654	1,071,922	1,075,771	1,078,405	1,071,028.50	5,634.04	0.53
5	781,822	780,363	784,140	784,832	788,171	786,933	784,376.83	2,960.78	0.38
6	673,755	672,136	675,124	676,588	677,178	678,210	675,498.50	2,273.61	0.34
7	863,661	862,051	866,539	868,047	869,952	870,160	866,735.00	3,323.52	0.38
8	734,205	732,343	736,626	737,577	739,668	739,408	736,637.83	2,901.80	0.39

[a]Regaloside K (1), regaloside C (2), regaloside H (3), regaloside A (4), regaloside F (5), regaloside E (6), regaloside B (7), and regaloside I (8).

Table S4

System suitability for simultaneous analysis of the eight regalosides by HPLC method.

Analyte[a]	Parameter[b]				
	k′	α	*N*	*Rs*	*S*
1	3.90	1.18	266830.64	11.02	1.02
2	4.58	1.06	400274.99	4.59	1.04
3	4.86	1.06	403043.59	4.59	1.03
4	5.60	1.08	529454.68	7.65	1.03
5	6.07	1.08	586865.43	7.65	1.08
6	6.79	1.12	738662.12	11.61	1.04
7	8.00	1.03	870983.48	3.12	1.02
8	8.21	1.03	866388.64	3.12	1.01

[a]Regaloside K (1), regaloside C (2), regaloside H (3), regaloside A (4), regaloside F (5), regaloside E (6), regaloside B (7), and regaloside I (8).
[b]Parameters: *k′*; retention factor, α; selectivity factor, *N*; theoretical plate number, *Rs*; resolution, and *S*; symmetry factor

Table S5

IC_{50} values of BLL extract and the eight regalosides in ABTS and DPPH ROS scavenging assays.

Analyte	IC_{50} (µg/mL and µM)[a]	
	**ABTS**	**DPPH**
BLL extract	942.8 ± 12.0	813.7 ± 4.2
Regaloside K	192.6 ± 21.8	66.1 ± 4.5
Regaloside C	139.0 ± 5.2	51.6 ± 3.0
Regaloside H	>400	>400
Regaloside A	>400	>400
Regaloside F	>400	104.5 ± 16.1
Regaloside E	121.1 ± 2.1	46.6 ± 2.6
Regaloside B	>400	>400
Regaloside I	>400	>400
Ascorbic acid[b]	108.2 ± 14.6	50.7 ± 0.1

All data are expressed as mean ± SD.

[a]Concentration (µg/mL and µM) required for 50% reduction of activity.

[b]Ascorbic acid was used as a positive control for ABTS DPPH ROS scavenging activities.

Table S6

Information on the eight regalosides selected as a marker compound for quality control of BLL.

Analyte[a]	Purity (%)	Molecular formula	Molecular weight	CAS No.	PubChem CID	Catalog No.	Maker
1	91.1	$C_{18}H_{24}O_{11}$	416.4	13872-00-6	-	BD-R1387	BioFron
2	99.2	$C_{18}H_{24}O_{11}$	416.4	117591-67-6	14135348	DR13384	Shanghai Sunny Biotech
3	96.2	$C_{18}H_{24}O_{10}$	400.4	126239-77-8	14542288	DR13046	Shanghai Sunny Biotech
4	98.1	$C_{18}H_{24}O_{10}$	400.4	114420-66-5	5459131	DR13382	Shanghai Sunny Biotech
5	100.0	$C_{19}H_{26}O_{11}$	430.4	120601-65-2	14284479	DR13039	Shanghai Sunny Biotech
6	98.7	$C_{20}H_{26}O_{12}$	458.4	123134-21-4	163321613	TBW04018	Wuhan ChemNorm Biotech
7	98.2	$C_{20}H_{26}O_{11}$	442.4	114420-67-6	5459143	DR13383	Shanghai Sunny Biotech
8	98.4	$C_{20}H_{26}O_{11}$	442.4	126239-78-9	-	CFN95525	Wuhan ChemFaces Biochemical

[a]Regaloside K (1), regaloside C (2), regaloside H (3), regaloside A (4), regaloside F (5), regaloside E (6), regaloside B (7), and regaloside I (8).

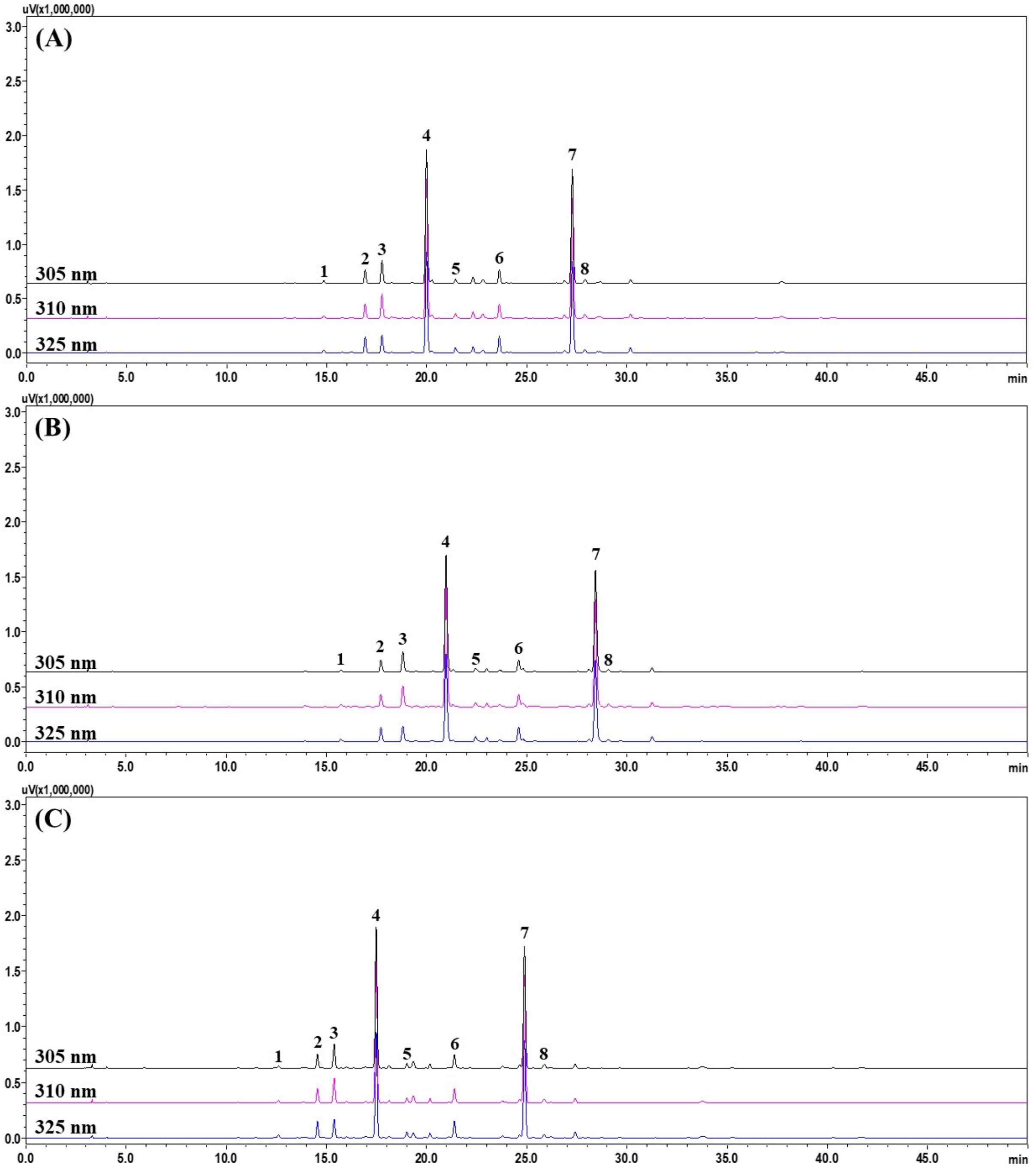

Figure S1. Comparison of HPLC chromatograms according to column type at a column temperature of 40°C and a mobile phase containing 0.1% (v/v) formic acid. A; Gemini C_{18} column. B; YMC-Triart C_{18} column. C; Hypersil GOLD C_{18} column. Regaloside K (1), regaloside C (2), regaloside H (3), regaloside A (4), regaloside F (5), regaloside E (6), regaloside B (7), and regaloside I (8).

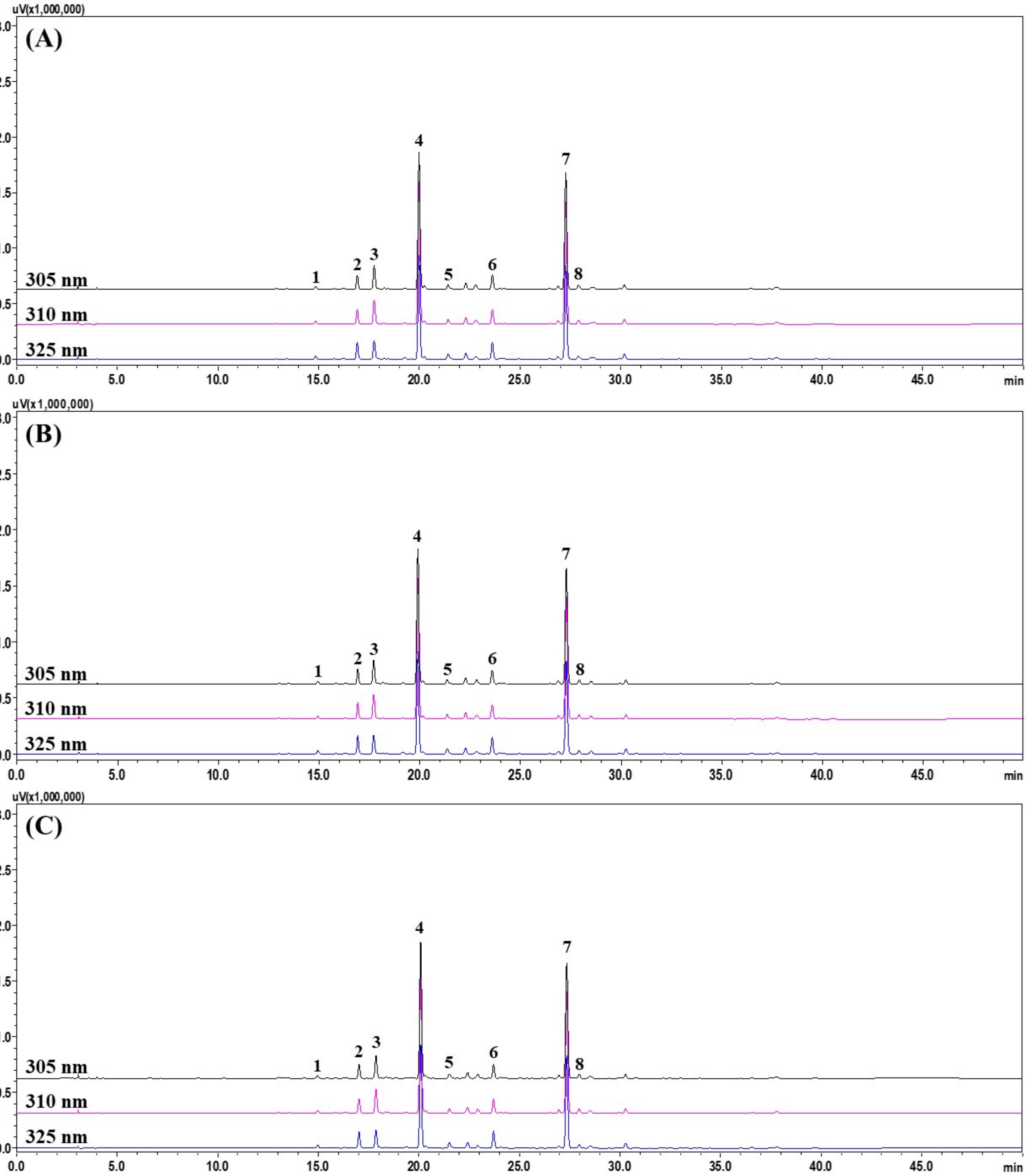

(A)
(B)
(C)
uV(x1,000,000)
305 nm
310 nm
325 nm
1
2
3
4
5
6
7
8
0.0
0.5
1.0
1.5
2.0
2.5
3.0
5.0
10.0
15.0
20.0
25.0
30.0
35.0
40.0
45.0
min

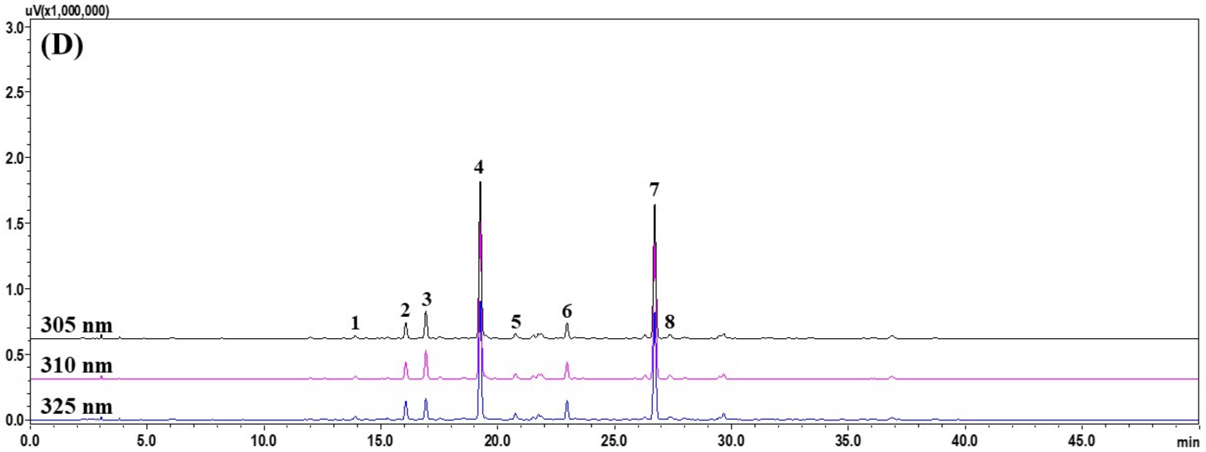

Figure S2. Comparison of HPLC chromatograms according to type of acid on a Gemini column maintained at 40°C. A; 0.1% (v/v) Formic acid, B; 0.1% (v/v) trifluoroacetic acid, C; 0.1% (v/v) phosphoric acid, and D; 1.0% (v/v) acetic acid. Regaloside K (1), regaloside C (2), regaloside H (3), regaloside A (4), regaloside F (5), regaloside E (6), regaloside B (7), and regaloside I (8).

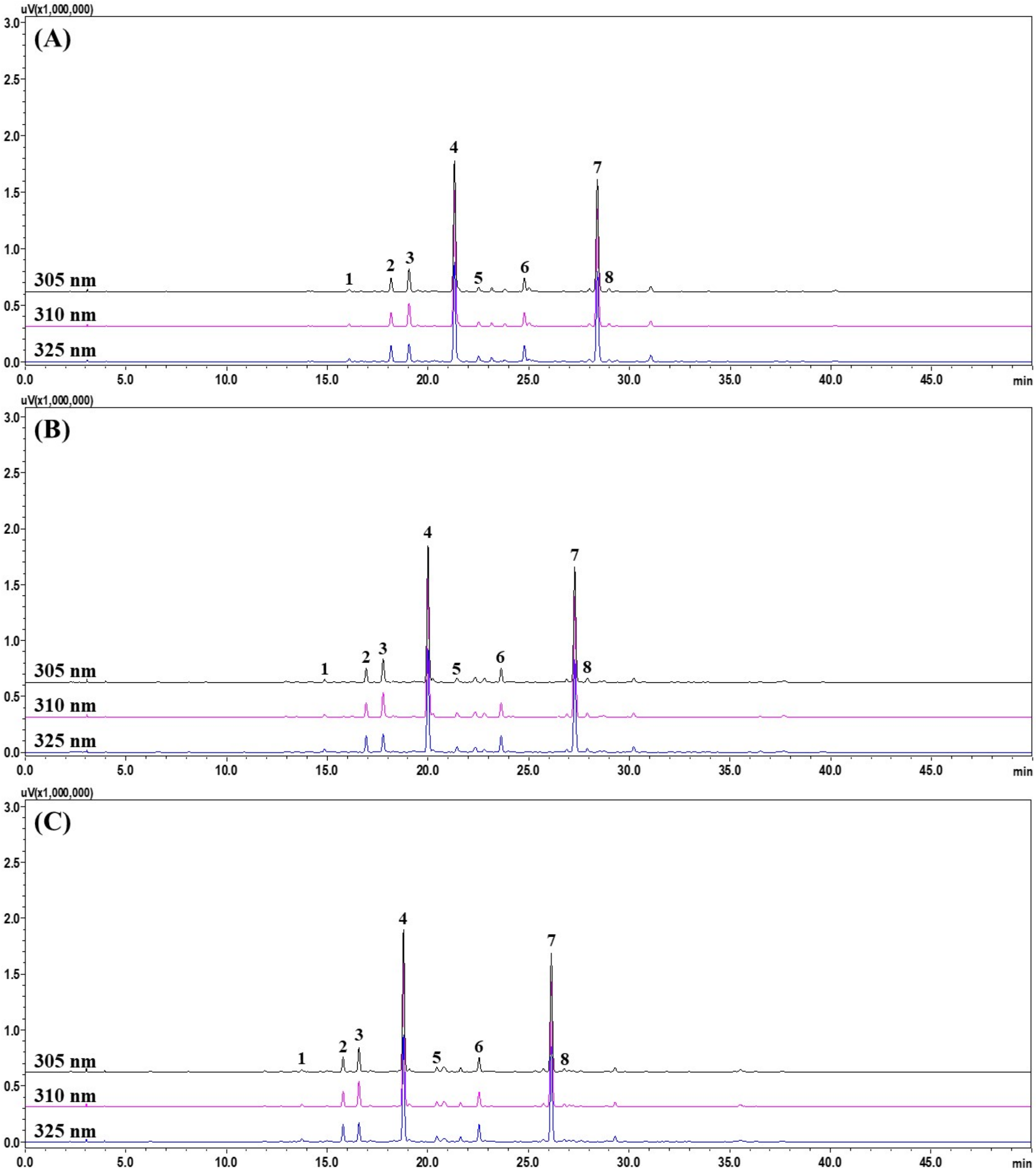

Figure S3. Comparison of HPLC chromatograms according to column temperature on a Gemini column and a mobile phase containing 0.1% (v/v) formic acid. A; 30 °C, B; 40 °C, and C; 50 °C. Regaloside K (1), regaloside C (2), regaloside H (3), regaloside A (4), regaloside F (5), regaloside E (6), regaloside B (7), and regaloside I (8).

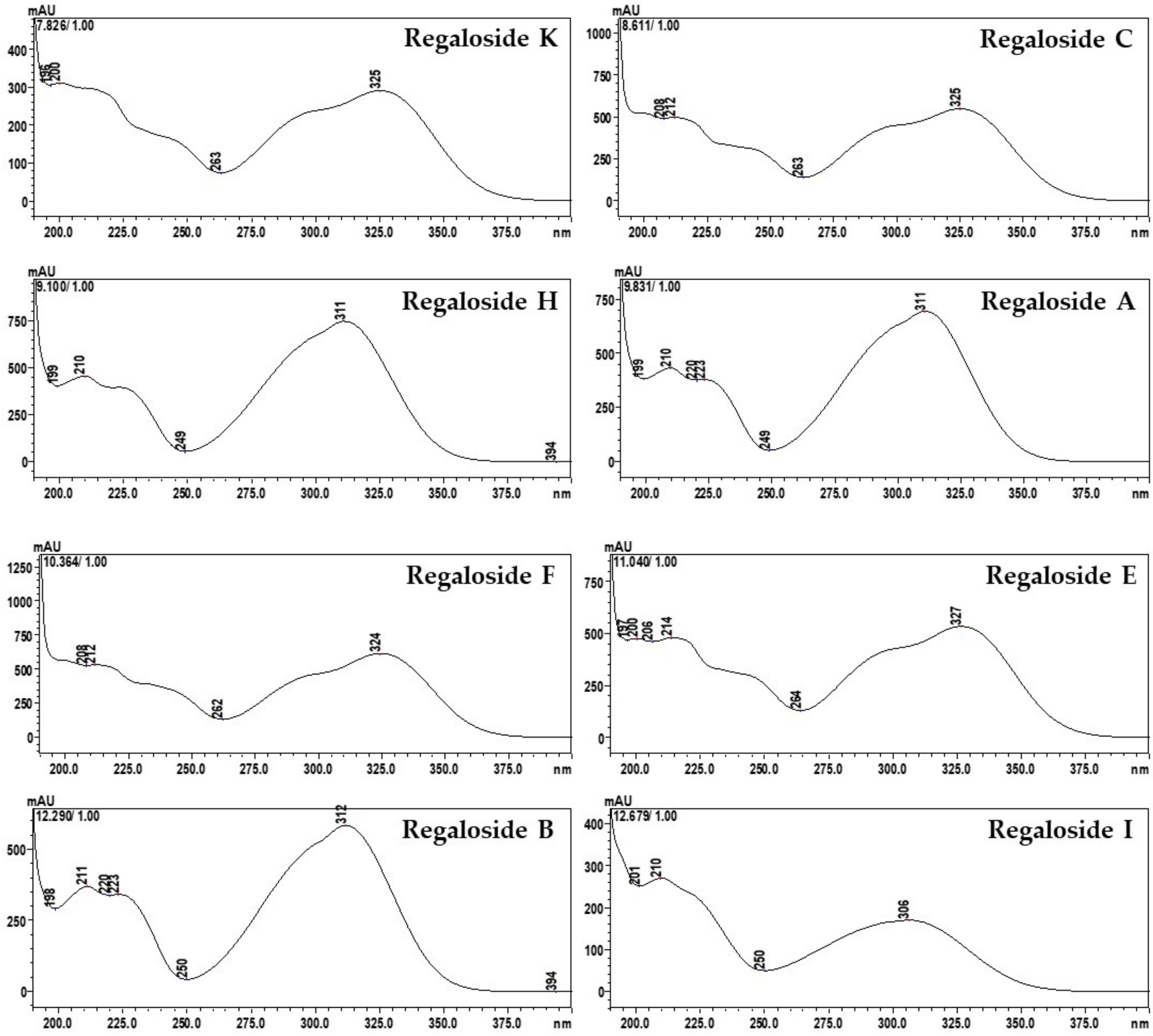

Figure S4. UV spectra of each regaloside.

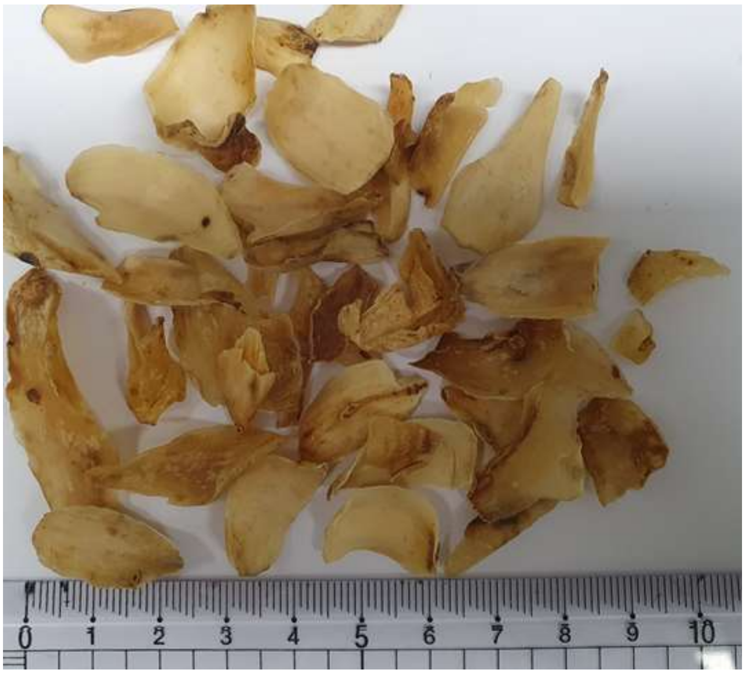

Figure S5. BLL used in the study
